# Discontinuing adalimumab in patients with controlled juvenile idiopathic arthritis-associated uveitis (ADJUST—Adalimumab in Juvenile Idiopathic Arthritis-associated Uveitis Stopping Trial): study protocol for a randomised controlled trial

**DOI:** 10.1186/s13063-020-04796-z

**Published:** 2020-10-27

**Authors:** Nisha R. Acharya, Caleb D. Ebert, Nicole K. Kelly, Travis C. Porco, Athimalaipet V. Ramanan, Benjamin F. Arnold, the ADJUST Research Group, Nisha R. Acharya, Travis C. Porco, Benjamin F. Arnold, Thuy Doan, John A. Gonzales, Emily von Scheven, Shelia T. Angeles-Han, Virginia Miraldi Utz, Albert T. Vitale, Aimee O. Hersh, Rebecca S. Overbury, Erin D. Stahl, Ashley M. Cooper, Melissa A. Lerman, Stefanie L. Davidson, Jennifer E. Thorne, Ekemini A. Ogbu, Kabita Nanda, Erin P. Herlihy, Michelle T. Cabrera, Mark S. Dacey, Katharine F. Moore, Catherine Guly, Andrew D. Dick, Harry Petrushkin, Elena Moraitis, Ameenat L. Solebo, Gavin Cleary, Jose A. Gonzalez-Martin, Sharmila Jandial, Michael P. Clarke, Kristina May, Alice Leahy, Jessy Choi, Daniel Hawley, Narman Puvanachandra, Kate Armon, William D. Renton, Robyn Troutbeck

**Affiliations:** 1 F. I. Proctor Foundation, University of California, San Francisco, 513 Parnassus Avenue, San Francisco, CA 94143 USA; 2 Department of Ophthalmology, University of California, San Francisco, 513 Parnassus Avenue, San Francisco, CA 94143 USA; 3 Department of Epidemiology and Biostatistics, University of California, San Francisco, USA; 4 Bristol Royal Hospital for Children, Upper Maudlin Street, Bristol, BS2 8BJ UK

**Keywords:** Uveitis, Juvenile idiopathic arthritis, Adalimumab, Randomised controlled trial

## Abstract

**Background:**

Juvenile idiopathic arthritis (JIA)-associated uveitis is a chronic paediatric ocular inflammatory condition that can result in visual impairment. Adalimumab, a tumour necrosis factor (TNF)-alpha inhibitor, effectively controls joint and eye inflammation; however, its long-term use may increase the risk of adverse health outcomes and place an undue financial burden on the patient and healthcare system given its high cost. There is great interest for patients to stop adalimumab following remission due to these reasons but there is a lack of information on the ability to maintain control after discontinuing adalimumab.

**Methods:**

The Adalimumab in Juvenile Idiopathic Arthritis-associated Uveitis Trial (ADJUST) is a multicentred, international trial that will randomise 118 participants aged 2 years and older with controlled JIA-associated uveitis to either continue adalimumab or discontinue adalimumab and receive a placebo. The trial will compare the time to uveitis recurrence between the two groups over 12 months. All participants will receive the standard weight-based dose of adalimumab or placebo: 20 mg biweekly (if < 30 kg) or 40 mg biweekly (if ≥ 30 kg).

**Discussion:**

This is the first randomised controlled trial to assess the efficacy of discontinuing adalimumab after demonstrating control of JIA-associated uveitis for at least 12 months. The results of ADJUST will provide information on clinical outcomes to guide clinicians in their decision-making regarding discontinuation of adalimumab.

**Trial registration:**

ClinicalTrials.gov NCT03816397. Registered on 25 January 2019. EudraCT 2019-000412-29. Registered on 17 January 2019

## Administrative information

**Table:** 

Title {1}	Discontinuing adalimumab in patients with controlled juvenile idiopathic arthritis-associated uveitis (ADJUST – Adalimumab in Juvenile Idiopathic Arthritis-associated Uveitis Stopping Trial): study protocol for a randomised controlled trial
Trial registration {2a and 2b}.	ClinicalTrials.gov, NCT03816397. Registered on 25 January 2019. EudraCT, 2019-000412-29. Registered on 17 January 2019.
Protocol version {3}	Version 1.2; 10 April 2020
Funding {4}	National Eye Institute, National Institutes of Health, UG1 EY029658
Author details {5a}	^1^F. I. Proctor Foundation, University of California, San Francisco ^2^Department of Ophthalmology, University of California, San Francisco ^3^Department of Epidemiology and Biostatistics, University of California, San Francisco. ^4^Department of Paediatric Rheumatology, Bristol Royal Hospital for Children, Upper Maudlin Street, Bristol, UK
Name and contact information for the trial sponsor {5b}	Nisha Acharya, MD, MS, University of California, San Francisco, San Francisco, CA, 94143, USA
Role of sponsor {5c}	Nisha Acharya is the Principal Investigator for the trial and was responsible for the trial design, writing of this report, and decision to submit the report for publication. The funding agency, the National Institutes of Health, had no direct role in the design of the trial, will not have authority over the collection, management, analysis, nor interpretation of data, and was not a part of the decision to write and submit this report for publication.

## Introduction

### Background and rationale {6a}

Juvenile idiopathic arthritis (JIA) is the most common paediatric rheumatological condition with an estimated prevalence of 3.8 to 400 per 100,000 children [1]. Upwards of one third of patients diagnosed with JIA develop uveitis—a chronic, asymptomatic, anterior inflammation of the eye—within 7 years of disease onset [2–4]. There is significant evidence of poor long-term visual outcomes for JIA-associated uveitis, with one third of all patients developing substantial visual impairment and 10–15% becoming legally blind [3, 5, 6].

Topical steroids are the first-line treatment for JIA-associated uveitis, yet there are risks associated with their long-term use (e.g., development of cataracts), and many patients are refractory to this treatment alone [7]. Conventional disease-modifying anti-rheumatic drugs (DMARDs) and biologic DMARDs have been demonstrated to be effective for controlling inflammation in JIA-associated uveitis. Biologics in particular have largely transformed the management of JIA-associated uveitis, and adalimumab, one of the primary biologic DMARDs, has been greatly efficacious for this indication. Adalimumab is a recombinant human IgG1 monoclonal antibody specific for tumour necrosis factor (TNF)-alpha that has been shown to effectively control inflammation in more than 80% of JIA-associated anterior uveitis cases that are refractory to methotrexate [8]. Adalimumab is approved by the US Food and Drug Administration (FDA) in children with polyarticular JIA and non-infectious intermediate, posterior, and panuveitis; by the UK National Health Service (NHS) in children with paediatric chronic anterior uveitis and JIA; and by the Australian Therapeutic Goods Administration (TGA) for polyarticular JIA [9–11].

While adalimumab has successfully been used to treat JIA-associated uveitis, there are safety concerns and financial implications associated with its long-term use. Adalimumab, like other TNF-alpha inhibitors, is associated with an increased risk of opportunistic infections and the development of immune-mediated resistance to the drug [12–16]. There is also a black box warning with respect to the risk of malignancy [9]. Additionally, adalimumab has a high financial cost to patients and society. In 2015, the International Federation of Health Plans reported that two pre-filled syringes—a 28-day supply—cost $2669 in the US (without insurance) and $1362 in the UK [17]. Beyond the direct cost of the medicine, there are many hidden costs of adalimumab therapy, including productivity loss and the psychological burden of administering injections that may cause systemic side effects.

Due to its high cost, insurance coverage for adalimumab varies across the US, and the UK NHS encourages discontinuation among children who respond well to adalimumab therapy. However, no prospective studies have demonstrated whether or not discontinuing adalimumab is safe for children. To date, withdrawal of immunomodulatory therapies for JIA-associated uveitis and other autoimmune diseases has been restricted to retrospective case studies and observational cohorts. These limited studies have demonstrated a high relapse rate within 12 months of discontinuation. In a cohort of 335 children with polyarticular JIA or enthesitis-related arthritis, 89% of participants who withdrew from anti-TNF-alpha therapy (while remaining on methotrexate) experienced a recurrence of inflammation within 12 months [18]. Smaller cohort studies have shown similar results: discontinuing infliximab in participants with JIA-associated uveitis resulted in a median time to recurrence of 76 days, and in another cohort, the median time to relapse in JIA participants who stopped etanercept was 4.3 months [19, 20].

The Adalimumab in Juvenile Idiopathic Arthritis-associated Uveitis Stopping Trial (ADJUST) is the first clinical trial to examine the safety and efficacy of stopping adalimumab in participants with controlled JIA-associated uveitis. The trial will also collect clinical and laboratory predictors of uveitis relapse and assess rates of achieving control of uveitis after restarting treatment. Data from this trial will provide an evidence base to inform treatment recommendations on the use of adalimumab in patients with JIA-associated uveitis.

## Objectives {7}

The study has three primary aims:

1. To compare time to the recurrence of ocular inflammation in JIA-associated uveitis participants who continue adalimumab versus those who discontinue adalimumab;
2. To determine predictors of recurrence of JIA-associated uveitis; and
3. To determine if stopping the use of adalimumab results in worse overall control of inflammation after 6 and 12 months, including among participants who experience treatment failure and re-initiate adalimumab use.

## Trial design {8}

**Fig. 1:**
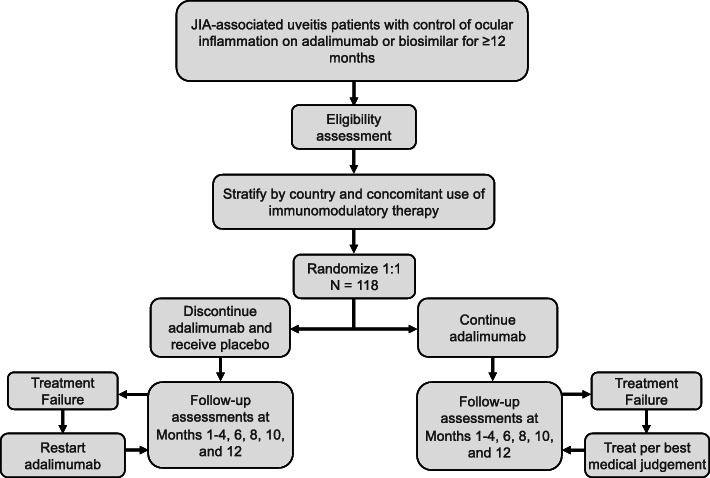
Participant flowchart

ADJUST is an international, multicentre, randomised, double-masked, phase IV superiority clinical trial comparing the time to recurrence of ocular inflammation between JIA-associated uveitis patients with control of inflammation who discontinue adalimumab to those who continue adalimumab treatment. Randomisation will be stratified by country and use of a concomitant immunomodulatory agent (i.e., methotrexate, mycophenolate mofetil, azathioprine, and leflunomide), with equal allocation within strata (Fig. 1).

## Methods: participants, interventions, and outcomes

### Study setting {9}

The trial will take place across the US, the UK, and Australia. All study sites have an established paediatric rheumatology-ophthalmology clinic or a close working relationship between these subspecialty clinics. Additionally, the clinics in the UK are a part of a uveitis-paediatric rheumatology network that has a strong record of participation in JIA and JIA-associated uveitis clinical trials.

### Eligibility criteria {10}

Participants aged 2 years and older who have been diagnosed with JIA-associated uveitis (with no other suspected aetiology) and have been on adalimumab (or a biosimilar of adalimumab) for at least 12 consecutive months with demonstrated control of ocular inflammation (≤ 0.5+ anterior chamber (AC) cell, ≤ 0.5+ vitreous haze, and no active retinal/choroidal lesions in either eye and no macular oedema) and arthritis are eligible for participation in the trial. Additionally, participants must meet the following eligibility criteria:

- History of JIA diagnosed prior to 16 years of age;
- Been on a stable dose of adalimumab (or biosimilar of adalimumab) for the last 180 days, and the dose must not exceed the standard weight-based biweekly dose of 20 mg (if the participant weighs < 30 kg) or 40 mg (if the participant weighs ≥ 30 kg);
- If on concomitant antimetabolite therapy, the antimetabolite (injectable or oral methotrexate, mycophenolate mofetil, azathioprine, or leflunomide) dose must have been stable for the last 90 days and cannot exceed the maximum allowable dose;
- If on topical corticosteroids, the topical corticosteroid has been stable for the last 90 days, and it does not exceed 2 drops per day of prednisolone acetate 1% or equivalent;
- Willingness to comply with all study procedures and availability for the duration of the study period;
- Willingness to limit consumption of alcohol during the study period;
- Agreement to avoid live attenuated vaccinations;
- Agreement to use effective contraception or abstinence for ≥ 28 days prior to screening and throughout the study period (for patients of reproductive age);
- A negative tuberculosis (TB) test within the past 12 months or a positive test for TB with a history of completed treatment for latent TB;
- Suitable, in the opinion of the investigator, to continue treatment with adalimumab or placebo per regional labelling; and
- No contraindications to receive adalimumab as per the local Summary of Product Characteristics.

Any of the following will exclude a participant from enrolling in the trial:

- History of acute anterior uveitis characterised by redness and symptoms including but not limited to floaters, pain, and light sensitivity;
- Intraocular surgery in the past 90 days or planned surgery in the next 12 months;
- Severe cataract or opacity preventing view to the posterior pole in both eyes;
- Chronic hypotony in either eye;
- Treatment with oral corticosteroids within the last 12 months (oral corticosteroids are permitted if prescribed for conditions unrelated to JIA (e.g., allergic reaction, asthma) and not anticipated to be needed again during the trial);
- Intraocular corticosteroid injections within the last 12 months;
- Use of non-steroidal anti-inflammatory drug (NSAID) eye drops within the last 90 days;
- Pregnancy or lactation;
- Prior safety or tolerability issues with adalimumab;
- History of cancer, active tuberculosis, or hepatitis B;
- Other medical conditions expected to dictate treatment course during the trial; or
- Abnormal lab values within 90 days prior to enrolment.

### Who will take informed consent? {26a}

All participants who are at least 16 years of age in the UK and Australia and at least 18 years of age in the US will provide written, informed consent. Participants who are at least 7 years of age but less than 16 in the UK and Australia or 18 in the US will provide written assent for the trial. These participants must also have a parent or guardian provide written, informed consent to enrol in the trial. Participants younger than 7 years of age only require a parent or guardian to provide written, informed consent on their behalf.

A member of the local research team will hold a discussion with the participant and their parent/guardian (if a minor) to review trial participation and the risks and benefits of the trial. The team member will ensure that the participant understands the trial and will answer any questions. Assent/consent will be recorded by obtaining a signature, and participants will receive a copy of the informed consent.

### Additional consent provisions for collection and use of participant data and biological specimens {26b}

Participants will be asked to provide additional specimens: blood samples (for a biobank) and rectal swab samples. Specimen collection is an optional component of the trial, and participants may decline either or both samples and still enrol in the trial.

## Interventions

### Explanation for the choice of comparators {6b}

The primary outcome is the time to recurrence of inflammation, also known as treatment failure. This definition of treatment failure is based on the grading of ocular inflammation and classification of arthritis, both of which are subjective. Given the subjectivity of the primary outcome and the possible biases that investigators, participants, and their family/caregivers may hold regarding adalimumab, a placebo will be used to mask all personnel involved in patient care. The placebo looks and feels identical to the pre-filled syringes containing active adalimumab.

### Intervention description {11a}

Regardless of the randomisation group, each participant will subcutaneously inject the study medication biweekly using pre-filled syringes with the standard weight-based dose of 20 mg if the participant weighs < 30 kg and 40 mg if the participant weighs ≥ 30 kg. Both groups will be followed for 12 months.

Open-label adalimumab will be provided to all participants who experience treatment failure if the decision is made to treat with adalimumab post-treatment failure, regardless of their randomisation group. Participants who were randomised to discontinue adalimumab and inject the placebo are highly encouraged to restart adalimumab following treatment failure.

### Criteria for discontinuing or modifying allocated interventions {11b}

Participants who develop a fever of at least 101 °F/38.3 °C or an infection requiring antibiotics should consult their investigator regarding treatment; these participants may stop taking the study medication and concomitant antimetabolite per the investigator’s discretion. Additionally, the study medication and antimetabolite should be immediately stopped in the event of lab abnormalities, a pregnancy, and/or adverse events. Participants should resume their treatment as soon as it is deemed safe to do so by the investigator. The study medication should not be changed except if the participant’s weight reaches the higher dose threshold (≥ 30 kg) and is sustained for 2 consecutive study visits at least 28 days apart.

### Strategies to improve adherence to interventions {11c}

All participants who enrol in the trial will have previously received biweekly injections of adalimumab (or a biosimilar of adalimumab). While some participants may be on a less frequent schedule prior to enrolment, all participants will have experience adhering to a biweekly injection schedule. To assist with adherence, participants will be provided with a diary to record when injections were administered and to record their antimetabolite therapy adherence, if applicable.

### Relevant concomitant care permitted or prohibited during the trial {11d}

Participants are permitted to be on a concomitant antimetabolite (injectable or oral methotrexate, mycophenolate mofetil, azathioprine, or leflunomide) if the dose does not exceed the maximum allowable dose (methotrexate, 25 mg/week; mycophenolate mofetil, 3 g/day; azathioprine, 250 mg/day; leflunomide, 20 mg/day), and the dose remains consistent throughout the trial. Topical corticosteroids are permitted if the dose is no greater than 2 drops daily of 1% prednisolone acetate (or equivalent) and remains stable throughout the trial or until treatment failure is declared. Cycloplegic drops may be added if the participant has active inflammation and is at risk for synechiae. Intraocular pressure-lowering (IOP) drops are always allowed. Oral NSAIDs and local corticosteroid injections to the joints are permitted and are the preferred treatment if joint inflammation recurs. Systemic corticosteroids (unless prescribed for indications not related to the participant’s JIA-associated uveitis), intraocular corticosteroid injections, and NSAID eye drops are not permitted prior to treatment failure. There are no restrictions to concomitant care after treatment failure.

### Provisions for post-trial care {30}

All participants will be followed for at least 12 months. Participants that experience a treatment failure will be followed for at least 90 days after treatment failure. If not already unmasked, the local research team and the participant will be unmasked after completion of follow-up and the investigator will determine the best course of action for the participant’s medical treatment. No medication nor compensation will be provided to participants following the completion of the trial.

### Outcomes {12}

The primary outcome is time to treatment failure, with censoring at 12 months. Treatment failure is defined as the recurrence of ocular inflammation and/or the recurrence of joint inflammation that is persistent and severe enough to necessitate unmasking to manage the arthritis recurrence. A joint is defined to have active arthritis if the joint has swelling or at least two other signs of inflammation: heat, limited range of motion, tenderness, or painful range of motion. For treatment failure to be declared due to ocular recurrence, at least one of the following criteria must be present in at least one eye:

- ≥ 3+ AC cells at a single visit;
- ≥ 2-step increase from baseline AC cells at 2 separate visits at least 7 days apart;
- > 0.5+ AC cells at 2 separate visits at least 28 days apart;
- > 0.5+ vitreous haze at 2 separate visits at least 7 days apart; and/or
- Active retinal/choroidal lesions or macular oedema at 2 separate visits at least 7 days apart.

Time to treatment failure will be measured in days from the date of randomisation until the first date of increased ocular inflammation that was sustained for either 7 or 28 days (depending on the level of inflammation) or until the date when the rheumatologist declares that unmasking is necessary to manage the persistent and severe arthritis.

Secondary outcomes include the proportion of participants that experience a treatment failure, an arthritis flare, and macular oedema in each treatment group. Differences in best-corrected visual acuity (BCVA) scores, quality of life scores (from the Childhood Health Assessment Questionnaire [21], EuroQol 5 Dimension Youth Survey [22], Children’s Visual Functioning Questionnaire [23], and Effects of Youngster’s Eyesight on Quality of Life [24]), and Juvenile Arthritis Disease Activity Score (JADAS) values (JADAS-10 and JADAS-27 [25]) will also be compared between the randomised treatment groups. Other clinical indicators, including anti-drug antibodies (ADA) to adalimumab, serum MRP8/14 (calprotectin) levels, erythrocyte sedimentation rates (ESR), C-reactive protein levels (CRP), and gut microbiome biomarkers, will be explored as predictors for treatment failure.

### Participant timeline {13}

After participants are consented, their eligibility has been confirmed, and they have been randomised to either continue adalimumab or discontinue and receive the placebo, they will follow a 12-month study visit schedule. There are monthly ophthalmology study visits from baseline until month 4, followed by every other month until month 12. Rheumatology study visits will occur at baseline and months 3, 6, and 12. Standard-of-care laboratory samples will be collected every 3 months, and the research laboratory samples will be collected at baseline, month 6, and month 12. If a participant experiences treatment failure, then ophthalmology, rheumatology, and laboratory (standard-of-care and research samples) study visits will take place.

All participants will be followed for a minimum of 90 days after treatment failure occurs. If treatment failure is declared with fewer than 90 days remaining in the 12-month trial period, follow-up will be extended to allow for 90 days, where the maximum total length of follow-up is 15 months.

### Sample size {14}

A sample size of 118 participants (59 per group) provides 88% power to detect a hazard ratio of 2. We assumed an equal allocation between treatment groups and a 10% loss to follow-up throughout the study. The sample size was based on the approximate formula given in Freidman et al. for the number in each group [26]:

$$ N=\frac{{\left({Z}_{\alpha }+{Z}_{\beta}\right)}^2\left(\phi \left({\lambda}_C\right)+\phi \left({\lambda}_I\right)\right)}{{\left({\lambda}_I-{\lambda}_C\right)}^2} $$

where *λ* denotes the rate in the intervention arm (*λ*_*I*_) and control arm (*λ*_*c*_), *ϕ*(*λ*) = *λ*^2^/1 − *e*^−*λT*^ at censoring time *T*, and *Z*_*α*_ and *Z*_*β*_ are quantiles from the standard normal distribution at power (1 − *β*) and significance level *α*. A median time to treatment failure of 10 weeks in the group that discontinues adalimumab and 20 weeks in the group that continues adalimumab was conservatively estimated based on a series of retrospective studies. In two small retrospective studies, many JIA-associated uveitis patients who attempted to stop immunomodulatory therapies experienced a recurrence of inflammation within 100 days of discontinuing treatment [19, 20]. Another retrospective study specifically examined adalimumab discontinuation and found that participants experienced an ocular flare 3–7 months after stopping treatment [27].

### Recruitment {15}

Investigators at all enrolling clinics have extensive experience with the management of JIA-associated uveitis and have patient populations consistent with the trial’s target population. Details of the trial will be shared within investigators’ local networks and their larger professional organisations, as well as with patient- and parent-centred care support groups to leverage existing infrastructure and facilitate the identification of potential participants.

## Assignment of interventions: allocation

### Sequence generation {16a}

Participants will be randomised in a 1:1 ratio to masked adalimumab or masked placebo using small randomly permuted blocks. Randomisation will be stratified by country and use of antimetabolite medication. The allocation sequence for each country was generated by a trial statistician (BA) using R version 3.6.1 (R Project for Statistical Computing) [28].

### Concealment mechanism {16b}

Randomisation will occur using an online module within the Research Electronic Data Capture (REDCap) application employing the parameters described above. REDCap is a web-based application that is widely used for secure data collection and management practices [29, 30]. The Coordinating Center will upload the allocation sequence generated by the trial statistician to the REDCap module, where treatment assignments will be concealed from all study personnel until the time of randomisation. Study personnel will be unable to edit or access the full allocation sequence. Only researchers who have completed randomisation training and are certified by the Coordinating Center will have access to their country-specific REDCap randomisation module.

### Implementation {16c}

After the local research team has consented and enrolled a participant, the local treatment assigner will enter the participant’s ID, the date of randomisation, and whether or not the participant is on an antimetabolite into REDCap. REDCap will then generate the participant’s randomisation group.

## Assignment of interventions: blinding

### Who will be blinded {17a}

All personnel involved in patient care, including the participant and their parents/caregivers, will be masked to the randomised treatment assignment. The Principal Investigator and all ophthalmologists, rheumatologists, visual acuity examiners, optical coherence tomography operators, clinic coordinators, and refractionists will be masked. The pre-filled syringes with either adalimumab or placebo will look identical to ensure masking. The clinic pharmacists and treatment assigners will be unmasked to dispense the correct study medication.

### Procedure for unblinding if needed {17b}

Following the declaration of treatment failure, the investigator or coordinator will call the unmasked pharmacist and/or treatment assigner to unmask the entire local research team, the participant, and their parents/caregivers. Each clinic will have an emergency contact in case the pharmacist or treatment assigner is unavailable. In the event of an emergency where knowledge of the participant’s treatment assignment is essential to treat the participant, the investigator may willingly become unmasked following the procedures outlined above. However, the investigator should restrict the number of individuals who become unmasked when unmasking is not due to treatment failure.

## Data collection and management

### Plans for assessment and collection of outcomes {18a}

**Fig. 2:**
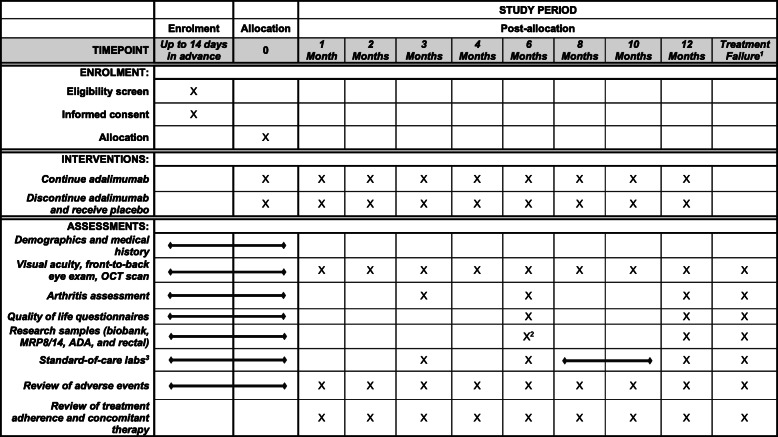
Schedule of enrolment, interventions, and assessments. Superscript number 1 indicates treatment failure can occur at any time. Superscript number 2 indicates rectal swab samples are not collected at month 6. Superscript number 3 indicates collected every 3 months

The primary outcome will be assessed at each study visit to determine whether or not the participant meets the criteria for treatment failure. Additional information regarding the participant’s characteristics, visual acuity, physical health, and quality of life will be collected at various study visits (see Fig. 2). Data will be collected directly on tablets using the REDCap Mobile App or, in the case of tablet failure, using either the web browser version of REDCap or identical paper-based study instruments. Study personnel who collect data will undergo training before trial initiation. All study instruments have undergone extensive testing to ensure the validity and reliability of the collected data.

### Plans to promote participant retention and complete follow-up {18b}

Given the eligibility criteria, the majority of participants will have been under the long-term care of the enrolling investigator and thus have an established relationship with the research team. Investigators are encouraged to only enrol participants who are expected to be highly compliant. Each clinic will collect contact information so that study visit reminders may be sent to participants; personal contact information will not be shared outside of the local research team. Each participant will also receive a trial card booklet as a resource to record future study visits. To further promote follow-up, participants will only receive enough study medication to last through the next study visit window, thereby requiring attendance at future visits.

### Data management {19}

Data will be entered directly into the REDCap database using encrypted tablets that require two separate login credentials—one for the tablet and one for the REDCap Mobile App. Each study instrument contains validation measures to ensure high-quality data collection: warnings will appear if data are entered outside of a pre-specified range or if fields remain unanswered prior to submission. Each clinic will only have access to their own participants’ data. Clinics will send data collected from the REDCap Mobile App to the Coordinating Center’s REDCap database after each study visit.

REDCap meets the highest standards for data security and compliance and is in accordance with the University of California, San Francisco’s (UCSF) data security policy. The data manager will regularly download the REDCap database and store the corresponding CSV files in a secure cloud-based folder, which also meets UCSF’s standards for data security. The data manager will routinely review the data for accuracy and completion, including range checks, missingness, and inconsistent or illogical entries. Additional details on data management procedures and practices can be found in the ADJUST Statistical Analysis Plan (SAP) and the Proctor Data Coordinating Center Handbook, both are available upon request to the Principal Investigator.

### Confidentiality {27}

Information will not be collected on participants who are screened but do not enrol in the trial, as informed consent is required prior to any data collection. Participants who provide consent and enrol will be assigned a unique, randomly generated ID to protect their privacy. The participant’s name and other identifying information will not be shared outside the local research team, including not being recorded in the REDCap database. Research data will be directly shared between the participant’s enrolling clinic and the ADJUST Coordinating Center. Representatives of the National Institutes of Health, the University of California, AbbVie, the National Health Service (for participants in the UK only), and the enrolling clinic’s institution may review research data for the purpose of monitoring the conduct of the trial, but they will not be provided with any personally identifiable information.

### Plans for collection, laboratory evaluation, and storage of biological specimens for genetic or molecular analysis in this trial/future use {33}

Participants will have serum drawn for MRP8/14, anti-adalimumab antibody and drug levels, and biobanking at the same time as their standard clinical laboratory blood draws. The MRP8/14 samples and biobanking samples will be stored at the participant’s local clinic in a − 80 °C freezer until the completion of the trial. The National Institute of Health Research Great Ormond Street (NIHR-GOSH) Biomedical Research Centre clinical immunology laboratory will receive and analyse all MRP8/14 samples. The biobank samples will be shared between the F.I. Proctor Laboratory at UCSF and the National Eye Institute’s Laboratory of Immunology. A range of approaches, including epigenetic, genetic, transcriptomic, and proteomic analyses, will be used to identify predictors of remission, uveitis recurrence, and responsiveness to treatment. The anti-adalimumab antibody and drug level samples will be sent immediately to the Exeter Clinical Laboratory at the Royal Devon and Exeter Hospital for analysis. Rectal swab samples will also be collected from participants and sent to the F.I. Proctor Laboratory at UCSF to evaluate changes in the gut microbiome and human transcriptomes using DNA and RNA deep sequencing techniques.

## Statistical methods

### Statistical methods for primary and secondary outcomes {20a}

The primary analysis will consist of a Cox proportional hazards model comparing time to treatment failure (measured in days from participant’s randomisation date) between treatment groups. Country and antimetabolite use will be included as fixed effects in the model. Hypothesis testing will be conducted using Monte Carlo permutation testing, and all tests will be two-sided with a type 1 error probability of alpha = 0.05. Analyses of secondary outcomes (e.g., BCVA scores, quality of life scores, macular oedema) will employ generalised linear models to estimate relative measures of association. The type of model (e.g., linear regression, log binomial regression) for these secondary outcomes will vary depending on the outcome of interest.

Secondary aims include identifying predictors of JIA-associated uveitis recurrence (aim 2) and evaluating if stopping adalimumab leads to overall less control of inflammation at 6- and 12-month visits even if participants restart adalimumab after a uveitis recurrence (aim 3). For aim 2, we will first use univariate log binomial regression, followed by multivariate analysis and model selection using the standard LASSO (least absolute shrinking and selection operator). The LASSO penalty will be estimated using 5-fold cross-validation. The variables selected from LASSO will be fit to the full dataset using logistic and log binomial regression. General elastic net regression will also be conducted to compare the results when using a ridge penalty to account for multicollinearity. For aim 3, we will use log binomial regression to model whether participants achieved corticosteroid-sparing control of ocular inflammation during the study. This analysis will follow the same template as the primary analysis described above. More detailed information regarding the planned statistical methods for the primary and secondary outcomes can be found in the ADJUST SAP, which can be made available upon request to the Principal Investigator.

### Interim analyses {21b}

ADJUST will be monitored by an independent Data and Safety Monitoring Committee (DSMC) appointed by the National Eye Institute. Regular in-person DSMC meetings will occur in year 1 and year 5 with teleconference calls at least every 6 months in between; however, the DSMC may receive trial results at any point upon request. Fully unmasked DSMC reports will be prepared by the study statistician per DSMC instructions and will include tabulated baseline covariates, all adverse events and serious adverse events (related and unrelated to study medication), and the primary outcome by treatment assignment and by site. Additional analyses required by the DSMC will be reported as needed. The DSMC has the authority to stop the trial for harm.

### Methods for additional analyses (e.g. subgroup analyses) {20b}

We plan to also conduct the primary and secondary analyses using a subgroup of participants who were randomised to discontinue adalimumab and later restarted adalimumab following treatment failure. Additional subgroup analyses are described in greater detail in the ADJUST SAP.

### Methods in analysis to handle protocol non-adherence and any statistical methods to handle missing data {20c}

The primary analysis will be conducted on an intent-to-treat basis where participants will be included based on their randomisation group regardless of adherence. Participants who are lost to follow-up will still be included in the analysis; their time will be censored after their last known study visit. A secondary per-protocol analysis may also be conducted as a sensitivity analysis.

While measures will be taken to minimise incomplete data, some missing values may exist. Analyses using missing covariates will not apply to the primary analysis as all individuals contribute observation time until censoring and no covariates are expected to be missing. However, secondary analyses may require the treatment of missing data. A complete case analysis will still be used for all secondary analyses and then multiple imputations will be performed as a sensitivity analysis to evaluate the impact of missing data on these secondary outcomes of interest.

### Plans to give access to the full protocol, participant-level data, and statistical code {31c}

ADJUST is registered on ClinicalTrials.gov (NCT03816397) and on the European Union Drug Regulating Authorities Clinical Trials Database (2019-000412-29). De-identified participant-level data and statistical code may be provided by request. The ADJUST Protocol and SAP may be made available upon request to the Principal Investigator.

## Oversight and monitoring

### Composition of the coordinating centre and trial steering committee {5d}

The ADJUST Coordinating Center is composed of faculty clinician-scientists, biostatisticians, and master-level researchers to provide oversight and implementation of the trial at all study sites. Responsibilities of the Coordinating Center include maintaining up-to-date human research approvals, conducting training and certification of all local research teams, supervising the trial activities at all sites, and monitoring safety, data quality, protocol adherence, and recruitment. The Principal Investigator will chair the Executive Committee that meets once a month to discuss overall trial progress, quality control issues, and any changes in study procedures, as necessary.

### Composition of the data monitoring committee, its role, and reporting structure {21a}

An independent DSMC has been appointed by the NIH. The composition of the DSMC includes biostatisticians, uveitis specialists, an ethicist, and a paediatric rheumatologist. The DSMC will meet approximately every 6 months, or more frequently if needed, to evaluate the trial’s progress and review any adverse events.

### Adverse event reporting and harms {22}

Adverse events will be collected at every study visit as well as 70 days after the participant’s last day of study participation and last dose of study medication. As specified by the US FDA, an adverse event will be considered serious if it results in any of the following: death, a life-threatening adverse event, inpatient hospitalisation or prolongation of existing hospitalisation, disability or permanent damage, or a congenital anomaly/birth defect [31]. If a serious adverse event occurs, the investigator will determine the relatedness of the serious adverse event to the study medication based on temporal relationship and clinical judgement. The degree of certainty about the association will be graded using the World Health Organization’s Uppsala Monitoring Centre categories: certain, probable, possible, or unlikely [32]. The investigator will also notify the appointed Medical Monitor and the trial sponsor of the serious adverse event. The Coordinating Center will notify AbbVie, the FDA, the Medicines & Healthcare products Regulatory Authority (MHRA), the UCSF Institutional Review Board, the South Central – Berkshire Research Ethics Committee (REC), the Royal Children’s Hospital Melbourne Human Research Ethics Committee (HREC), and the Therapeutic Goods Administration accordingly.

### Frequency and plans for auditing trial conduct {23}

The Coordinating Center will remotely evaluate the quality of trial activities on at least a monthly basis; however, quality control of trial data may be performed on a weekly basis. Weekly communication will be shared with the research group regarding trial progress and protocol adherence. In-person site visits will occur periodically to conduct chart reviews and train study personnel.

### Plans for communicating important protocol amendments to relevant parties (e.g. trial participants, ethical committees) {25}

All protocol amendments must first be approved by the NIH-appointed DSMC. Following the DSMC’s approval, amendments will be submitted to the UCSF IRB, the South Central – Berkshire REC, the Healthcare Research Authority (HRA), the MHRA (if applicable), and the Royal Children’s Hospital Melbourne HREC for approval. After approval from all local ethics boards, the amendment will be announced via a Protocol and Procedure Memorandum (PPM) that is delivered to all co-investigators and lead clinic coordinators.

## Dissemination plans {31a}

Most trial results will be communicated through peer-reviewed manuscripts. Results may also be shared at national and international conferences. Members of the research network and the Coordinating Center will primarily write the manuscripts and present results at conferences.

## Discussion

The Adalimumab in Juvenile Idiopathic Arthritis-associated Uveitis Stopping Trial is the first randomised controlled trial comparing continuing versus discontinuing adalimumab therapy in patients with controlled JIA-associated uveitis. While there is limited evidence of the safety and efficacy of discontinuing anti-TNF-alpha therapy, some health systems and health insurance agencies continue to enforce policies that require patients to discontinue or make it difficult to continue on therapy long term. In fact, the few published retrospective case series suggest that recurrence of inflammation may be common after discontinuing anti-TNF-alpha therapy [18–20]. ADJUST is a well-powered trial that will contribute to the evidence base for this understudied clinical question.

ADJUST will also address other key scientific questions. An important secondary aim is to explore factors that may predict JIA-associated uveitis relapse, including participant demographics, clinical characteristics, and novel biomarkers. These results may guide physicians when deciding which patients should discontinue adalimumab. Another aim of the trial is to identify if there is a risk of reduced responsiveness to adalimumab after restarting treatment following a failed discontinuation attempt. Studies on discontinuing other TNF-alpha inhibitors have raised concerns over a loss in drug efficacy once treatment is restarted [33, 34]. Reduced responsiveness can lead to worse ocular outcomes and thus is an important risk for physicians and patients to weigh when considering stopping therapy. Finally, the results from ADJUST may have an impact on healthcare policy and economics. Currently, there is variation in the coverage of adalimumab among healthcare systems and insurance companies, with some limiting the duration of use before requiring an attempt to discontinue therapy. There is no scientific evidence to support these practices.

One of the anticipated challenges of the trial will be recruitment. Participants are required to have at least 12 consecutive months of adalimumab treatment and control of their JIA-associated uveitis to be eligible for the trial. Many investigators have identified patients who are currently eligible; however, recruitment may become increasingly difficult following these initial enrolments as investigators wait for patients to meet the eligibility threshold. In anticipation of this challenge, a large number of recruiting centres across all regions of the US and the UK, as well as parts of Australia, have been included to provide a wide catchment area. All enrolling centres represent excellent, large centres of care that provide high-calibre paediatric rheumatology and uveitis care. In the event of significant enrolment challenges, the trial network will be expanded to include other institutions that have expressed interest in ADJUST.

There are many motivating factors to discontinue anti-TNF-alpha therapy after achieving control of inflammation, including the high cost of therapy, inconvenience of administering injections, and the risks of adverse events. However, there is a lack of evidence on the success and risks of attempting to do so in patients with JIA-associated uveitis. ADJUST aims to provide rigorous evidence on the safety and efficacy of this practice. The trial’s results will help guide the decision-making of physicians and their patients about whether discontinuing adalimumab is in their best interest.

## Trial status

The protocol reported here is version 1.2 dated 10 April 2020. Trial enrolment began on 3 March 2020 and will continue until at least 31 March 2023.

## Abbreviations

ADJUST: Adalimumab in Juvenile Idiopathic Arthritis-associated Uveitis Stopping Trial
BCVA: Best-corrected visual acuity
DSMC: Data and Safety Monitoring Committee
FDA: Food and Drug Administration
HRA: Health Research Authority
HREC: Human Research Ethics Committee
IRB: Institutional Review Board
JADAS: Juvenile Arthritis Disease Activity Score
JIA: Juvenile idiopathic arthritis
MHRA: Medicines & Healthcare products Regulatory Authority
NHS: National Health Service
NIH: National Institutes of Health
NSAID: Non-steroidal anti-inflammatory drug
OCT: Optical coherence tomography
PPM: Protocol and Procedure Memorandum
REC: Research Ethics Committee
SAP: Statistical Analysis Plan
SUN: Standardization of Uveitis Nomenclature
TGA: Therapeutic Goods Administration
TNF: Tumour necrosis factor
UCSF: University of California, San Francisco
UK: United Kingdom
US: United States
